# Relationship between visuospatial attention and paw preference in dogs

**DOI:** 10.1038/srep31682

**Published:** 2016-08-22

**Authors:** Marcello Siniscalchi, Serenella d’Ingeo, Serena Fornelli, Angelo Quaranta

**Affiliations:** 1 Department of Veterinary Medicine, Section of Behavioral Sciences and Animal Bioethics,University of Bari “Aldo Moro”, Italy

## Abstract

The relationship between visuospatial attention and paw preference was investigated in domestic dogs. Visuospatial attention was evaluated using a food detection task that closely matches the so-called “cancellation” task used in human studies. Paw preference was estimated by quantifying the dog’s use of forepaws to hold a puzzle feeder device (namely the “Kong”) while eating its content. Results clearly revealed a strong relationship between visuospatial attention bias and motor laterality, with a left-visuospatial bias in the left-pawed group, a right-visuospatial bias in the right-pawed group and with the absence of significant visuospatial attention bias in ambi-pawed subjects. The current findings are the first evidence for the presence of a relationship between motor lateralization and visuospatial attentional mechanisms in a mammal species besides humans.

It is well established that there is a complementary specialization of the two sides of the brain in terms of spatial attention, so that the right hemisphere processes information from the left visual field, and the left hemisphere processes information from the right visual field^1^,^2^. Attention deficit on the contralesional side of space following unilateral stroke (namely, unilateral spatial neglect) is a clear external manifestation of this phenomenon^3^,^4^.

However, left hemispatial neglect caused by damage to the right hemisphere occurs more than right hemispatial neglect due to left hemisphere stroke and asymmetries in recovery time show that right spatial neglect resolves more quickly than left (in other words, a right functionally-intact hemisphere can compensate for damaged left hemispheric spatial functions)^5^,^6^,^7^. Taken together, these findings supported the hypothesis of a right hemispheric advantage in the control of spatial attention resources^8^. Neuropsychological tests in healthy human subjects, such as the cancellation task, provide further evidence of right hemisphere superiority in spatial attention, reporting a systematic leftward bias during “cancellation” of visual items on a sheet of paper placed midline in front of them (i.e. “pseudoneglect” phenomenon)^9^,^10^.

A very similar leftward visuospatial bias was reported in a food detection task in which birds were required to explore an area in front of them and to sample grains^11^. Briefly, the leftward visuospatial bias was evident in both pecking activity and the order in which single pecks were made to the left and to the right-hand side of a surface uniformly spread with grains^11^.

Although preferential handedness is one of the striking features of motor control in humans^12^ and clear evidence exists that contralesional limb activation could reduce unilateral spatial neglect^13^,^14^, there are very few studies about how handedness may interact with spatial bias.

An effect of handedness on spatial perceptual biases has been recently reported in human studies^2^,^15^. FMRI analysis reported a right-lateralized brain network associated with attention system in right-handed but not in left-handed subjects^15^. Furthermore, during an auditory spatial localization task, Bareham *et al*.^2^ reported an opposite lateralized pattern of shift in attention associated with drowsiness in a population of 26 right-handed and 26 non right-handed healthy humans, suggesting that the relationship of handedness with hemispheric lateralization for attention is task-dependent.

The domestic dog may offer a valid animal model to study the relationship between motor lateralization and visuospatial attention mechanisms since the dog brain appears to be lateralized in a variety of perceptual sensory modalities (e.g. vision^16^,^17^, auditory^18^, olfaction^19^) and paw preference has been widely reported in different motor tasks^20^,^21^,^22^. In addition, paw preference in canine species has also been associated with functional differences at both behavioral and physiological levels^23^. Finally, it could be profitable to use canine species as a model to study the extent to which motor lateralized processes are related to visual attention, since dogs play a number of significant roles within the human community as workers during activities which demand spatial and motor skills (animal-assisted therapy, police work, security, and as guide dogs for visually-impaired humans).

In the light of such evidence, the aim of our research was to examine visuospatial attention lateralization in the canine species by presenting dogs with a food detection task that closely matches the cancellation task. Furthermore, we investigated the correlation between visuospatial bias and paw preference (evaluated by observing the use of the forepaws to handle a puzzle feeder device, namely the “Kong” test) to establish whether motor lateralization could be related to the development and control of spatial attention resources.

## Results

All the dogs started the experiment within the allowed time (2 minutes) and no behavioral signs of stress were manifested at any time during the experiment.

### Number of food items eaten in the left-right hemispace during the “cancellation” task

**Figure 1:**
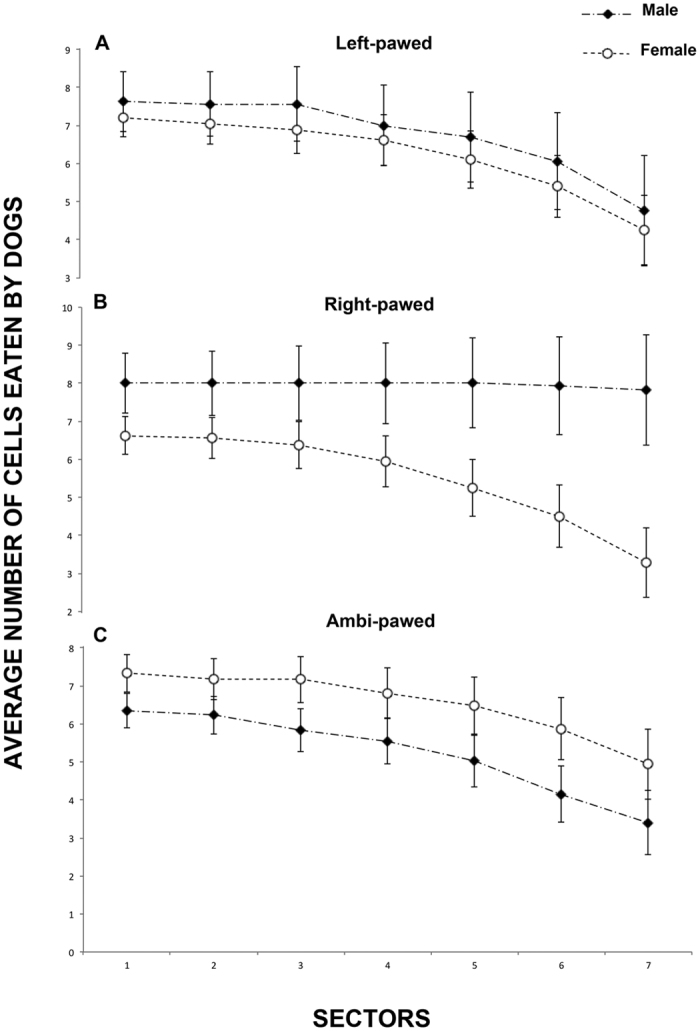
Average number of food items eaten by male and female dogs in the three behavioral categories. For the analysis, the surface of the Plexiglas board was divided into an array of 15 identical vertical sectors, with 7 sectors both to the left and right of the central midline sector. For each dog, all food items within each sector were counted. Data presented are means with S.E.M. calculated for each dog over the four trials.

Repeated-measures ANOVA analysis revealed a significant effect of distance on the number of food items eaten by the dogs (F(6, 114) = 84.431, P < 0.001), indicating that the amount of food items eaten lowered with distance from the centre (linear contrast: F(1, 19) = 87.206, P < 0.001) (see Fig. 1). A significant distance × sex (F(6, 114) = 2.815, P < 0.05) and distance × sex × paw preference (F(12, 114) = 5, 179, P < 0.001) interactions were revealed, indicating that male dogs tended to eat more items further from the centre than females and this was more evident for the right-pawed group (see Fig. 1).

**Figure 2:**
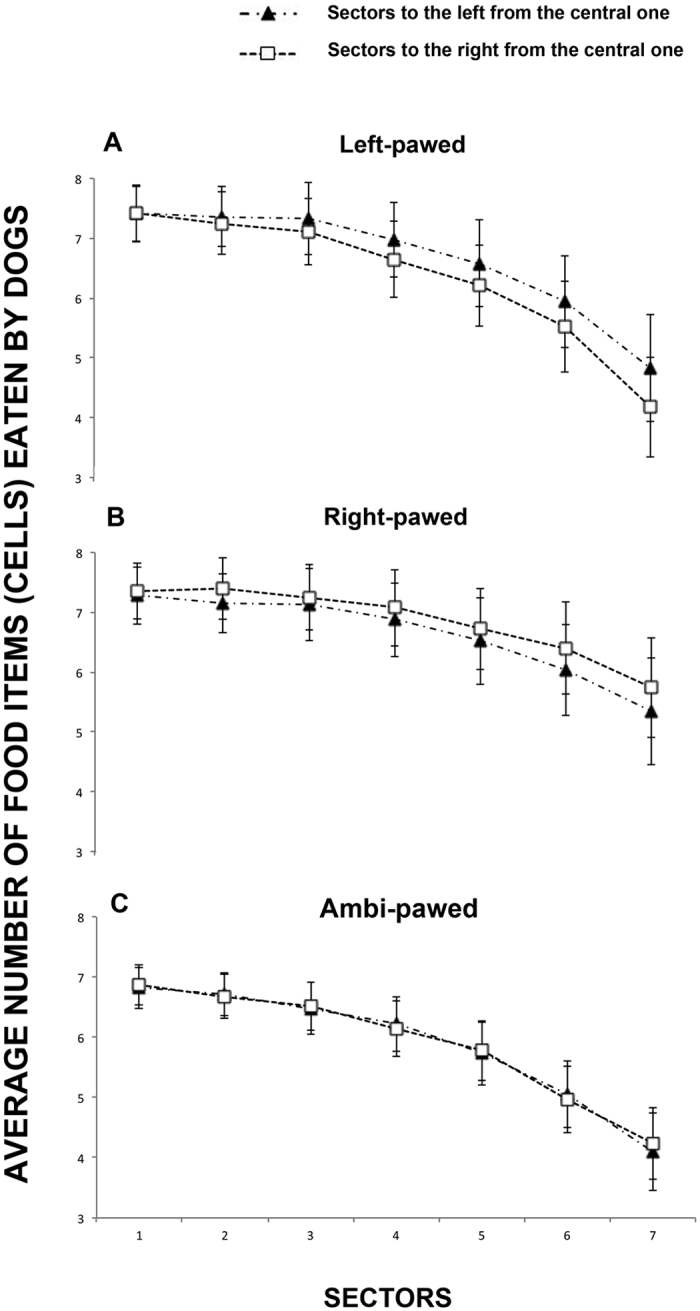
Average number of food items eaten by left-pawed, right-pawed and ambidextrous dogs. For the analysis, the surface of the Plexiglas board was divided into an array of 15 identical vertical sectors, with 7 sectors both to the left and right of the central midline sector. For each dog, all food items within each sector were counted. Data presented are means with S.E.M. calculated for each dog over the four trials.

**Figure 3:**
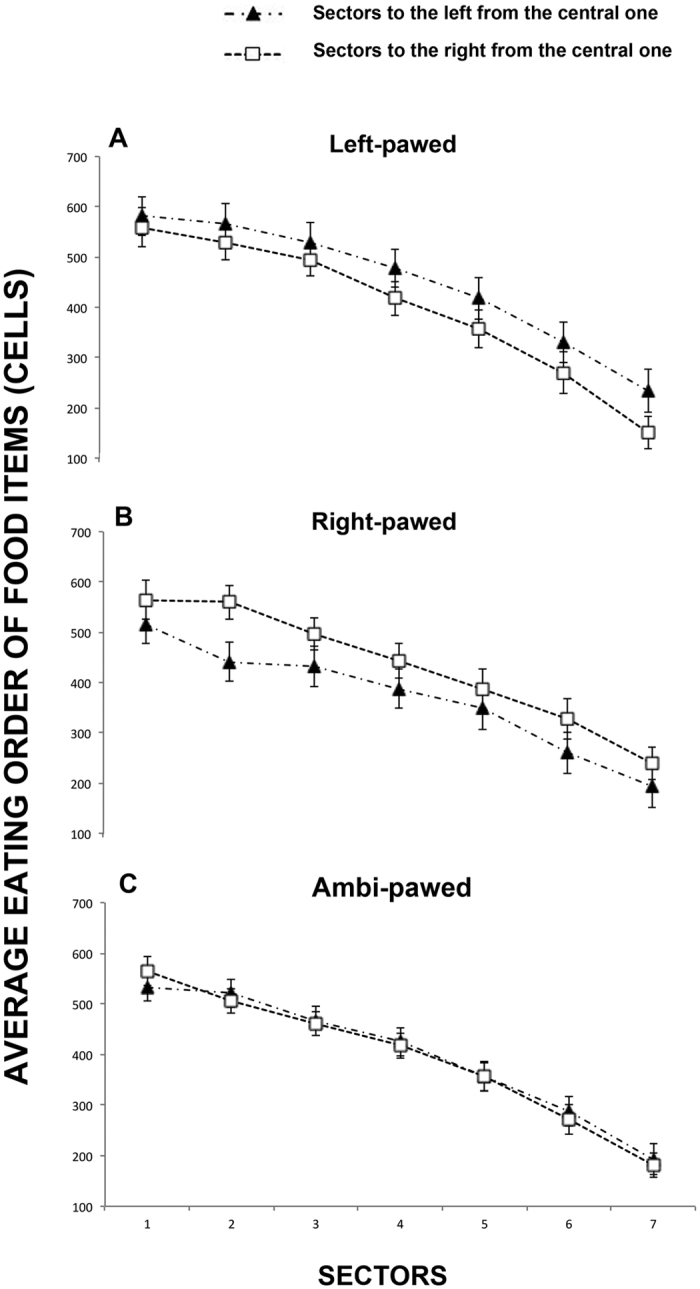
Average score for the order in which left-pawed, right-pawed and ambidextrous dogs ate food items in each sector. For the analysis, the surface of the Plexiglas board was divided into an array of 15 identical vertical sectors, with 7 sectors both to the left and right of the central midline sector. The spatial position of the food items eaten was scored and ranked based on the order in which they occurred. The location where dogs ate first received the highest score (112, as there were112 food items) while the last eating location received the lowest score of 1. These raw data were then analyzed considering the sequence with which food items were eaten at each left/right spatial position within each of the 7 sectors. Data presented are means with S.E.M. calculated for each dog over the four trials.

Although there was no significant left/right effect in the total number of food items eaten by the dogs during the cancellation task (sidedness: F(1, 19) = 0.185, P = 0.672), the results revealed a significant sidedness × paw-preference interaction (F(2, 19) = 10.195, P < 0.001) showing a significant rightward bias in right-pawed dogs (n = 7: Left = 6.62 ± 0.64, Right = 6.84 ± 0.62; m ± sem: t(6) = 3.708, P < 0.05), a significant leftward bias in left-pawed subjects (n = 7: Left = 6.63 ± 0.64; Right = 6.33 ± 0.62; m ± sem: t(6) = −2.581, P < 0.05) and no bias in ambi-pawed dogs (n = 11: Left = 5.87 ± 0.46, Right = 5.88 ± 0.45; m ± sem: t(10) = −0.286, P = 0.780) (see Fig. 2). Contrast revealed that the previously reported left/right effect becomes more evident with increasing distance from the centre (distance × sidedness × paw preference: F(12, 114) = 2.269, P < 0.05; see Fig. 3). Finally, ANOVA revealed a significant sidedness × sex interaction (F(1, 19) = 5.473, P < 0.05) showing a slight preference for male dogs to eat more food items located on the left-hand side with respect to the centre (Left = 6.70 ± 0.56; Right = 6.58 ± 0.54: t(8) = 1.041, P = 0.328) and for female dogs on the right side (Left = 6.04 ± 0.38; Right = 6.12 ± 0.37: t(15) = −1.015, P = 0.326).

No other statistically significant effects were apparent: paw preference (F(2, 19) = 0.689, P = 0.514); distance × paw preference (F(12, 114) = 1.516, P = 0.128); sex (1, 19) = 0.695, P = 0.415); paw preference × sex (F(2, 19) = 3.002, P = 0.074); sidedness × paw preference × sex (F(2, 19) = 1.473, P = 0.254); distance × sidedness (F(6, 114) = 0.406, P = 0.874); distance × sidedness × sex (F(6, 114) = 0.451, P = 0.843); distance × sidedness × sex × paw preference (F(12, 114) = 1.297, P = 0.230).

### Eating order of food items in the left-right hemispace during the “cancellation” task

Similarly, the effect of distance on the eating order of food items was significant (F(6, 114) = 227.085, P < 0.0001). Sectors close to the centre were chosen earlier than distant ones (linear contrast: F(1, 19) = 291.112, P < 0.001). A significant sidedness × paw preference interaction (F(2, 19) = 8.193, P < 0.001) indicated that left-pawed dogs showed a leftward bias in eating order (left = 439.54 ± 27.39, right = 392.97 ± 21.63: t(6) = 2.572, P < 0.05) while ambi-pawed dogs showed no left/right preference with regard to the eating order of food items (left = 401.27 ± 23.21, right = 395.46 ± 68.55: t(10) = 0.409, P = 0.691). A rightward trend on eating order was observed in the right-pawed group, though this was not statistically significant (left = 355.44 ± 41.06; right = 399.577 ± 45.04: t(6) = −1.755, P = 0.130) (see Fig. 3).

No other statistically significant effects were apparent: sex (F(1, 19) = 0.549, P = 0.468), paw preference (F(2, 19) = 0.233, P = 0.795), distance × paw preference (F(12, 114) = 0.712, P = 0.737), distance × sex (F(6, 114) = 1.265, P = 0.279), paw preference × sex (F(2, 19) = 2.101, P = 0.150), distance × paw preference × sex (F(12, 114) = 0.824, P = 0.626), sidedness (F(1, 19) = 0.064, P = 0.804), sidedness × sex (F(1, 19) = 3.272, P = 0.086), sidedness × paw preference × sex (F(2, 19) = 1.982, P = 0.165), distance × sidedness (F(6, 114) = 1.992, P = 0.072), distance × sidedness × paw preference (F(12, 114) = 1.409, P = 0.172), distance × sidedness × sex (F(6, 114) = 0.485, P = 818), distance × sidedness × paw preference × sex (F(12, 114) = 1.800, P = 0.056).

### Correlations between paw preferences and orienting attention laterality indices

Positive and statistically significant correlations were found between paw preferences and orienting attention laterality indices: LI _(paw preference)_ × LI _(Number of food items eaten)_ (r25 = 0.544, P = 0.004); LI _(paw preference)_ × LI _(Eating order of food items)_ (r25 = 0.414, P = 0.040); LI _(Number of food items eaten)_ × LI _(Eating order of food items)_ (r25 = 0.566, P = 0.003) (Pearson correlation), indicating that the paw preferentially used by the dogs during the Kong test was significantly related to the subjects’ orienting attention visual side.

In addition, repeated-measures ANOVA analysis revealed that there was no main effect of sessions on the two laterality indices (LI _(Number of food items eaten)_: (F(3, 72) = 0.655, P = 0.582); LI_(Eating order of food items)_: (F(3, 72) = 2.652, P = 0.055)), indicating that dogs were consistent in their performance across trials during the visual spatial task.

## Discussion

Lateralization of spatial attention has been reported in humans and birds that primarily attend to visual items in the left side of the space, suggesting right hemisphere superiority in the control of visuospatial function^8^,^11^. Here we report for the first time the presence of visuospatial lateralization in canine species, with different directions in relation to paw preference. The main results can be summarized as follows: dogs selected for their paw preference in a motor task requiring subjects to hold a food object (i.e. namely the Kong test) showed different visuospatial lateralization bias during a food detection task resembling the so-called cancellation test.

Left-pawed dogs exhibit a leftward bias in the total number of food items eaten from the testing apparatus (i.e. the Plexiglas board), a reversed rightward bias was observed in right-pawed subjects and no bias in ambidextrous dogs. This is intriguing, since it is the first evidence that clearly indicates a relationship between motor function and visuospatial bias in the animal kingdom, besides humans. The evidence of significant difference between the pawedness groups for the visuospatial food detection task is consistent with reports of a relationship between handedness and lateralization for spatial processes^24^,^25^,^26^, but is in contrast with other studies that have shown the lack of such a relationship^27^,^28^,^29^.

Nevertheless, in human fMRI studies, it has been reported that both attention network and spatial cognition are predominantly right hemisphere lateralized in right-handers but bilateral or even slight left-lateralized in non-right handers^15^,^25^. Here we found a reversed pattern in dogs. A possible explanation for this reversed pattern may emerge from a recent comparative study by Wells *et al*.^26^ who hypothesized that dogs, like humans, may use their non-dominant limb to stabilize the Kong ball and their dominant forelimb for postural support.

Since previous researchers in other animal models have shown that task type and complexity influence both the strength^30^,^31^ and degree^30^,^32^,^33^ of motor lateralization, more studies are required before definitive conclusions can be made. In dogs, different techniques have been used to determine motor lateral biases^20^,^21^,^22^,^34^,^35^; for example, removal of a blanket from over the head^22^, removal of tape placed over their nose^21^ or their eyes^35^, presentation of a paw on command^22^ and food retrieval from various devices^20^,^22^. Although motor lateralization results from both the Kong and Tape removal tests applied to the same population of dogs seem to be generally consistent between breeds, sexes and over time, differences between behavioral results from these two tests (i.e. a lack of consistency) suggest that motor lateralization is task-dependent even in canine species^36^. As a consequence, further research is required to verify whether the visuospatial biases reported here correlate with other expressions of canine motor laterality. However, it is interesting to note in the present work that the subjects’ motivation during both the visuospatial (the adapted version of the cancellation task) and the motor tasks (the Kong test) was very similar (i.e. food detection/retrieval) suggesting that motivation could also be a factor in lateralized visuospatial and motor biases. Data to support this hypothesis result from a previous study reporting that dogs’ visual motor bias to reach a target during a detour task was affected by subjects’ motivation to chase and capture a prey (i.e. prey-drive)^37^.

Furthermore, considering the eating order of food items, the significant effect of sidedness (left vs. right hemispace) was revealed only in the left-preferent behavioral category, which showed a clear leftward bias (right hemisphere activity).

This pattern is consistent with previous findings reporting a more reliable association between spatial abilities and sinistrality in human behavioral studies^27^ and with the more general hypothesis regarding right hemispheric superiority in the control of spatial attention resources^38^,^39^.

Another interesting aspect to consider is that canine forelimb attempts to reach the Kong are visually-guided movements. Experimental evidence from human studies have shown that visual attention in relation to forelimb movements (i.e. “motor attention”) and visuospatial “orienting attention” are distinct phenomena^40^,^41^. Indeed, it appears to be the neural structures located in the left hemisphere rather than in the right, that are dominant for motor attention^40^,^41^. In the light of this evidence if we consider a motor attention component in the Kong test, the fact that in canine species it is not lateralized in the left hemisphere (as it is in humans) but is related to an orienting attention function could support the hypothesis that, in humans, left hemisphere lateralization of motor attention could be a consequence of left hemisphere dominance for language. Nevertheless, the preferred paw used to stabilize the Kong ball was used predominantly also during contralateral attempts (i.e. when the puzzle feeder device was located contralaterally with respect to the dog’s visually preferred side, see supplementary materials), strengthening the fact that the asymmetries revealed by the Kong test are more likely to be motor rather than visual by their very nature.

In conclusion, dogs show a strong relationship between visuospatial orienting attention bias and paw preference related to food detection. Apart from contributing to our understanding of the evolution of brain lateralization in the animal kingdom, the very existence of such a relationship open the door to their exploitation in animal welfare, providing new evidence of the importance of a motor ability approach in order to help the rehabilitation of visual attention during pathological conditions (namely, unilateral spatial neglect). In addition, our findings have direct implications for canine species, not only because such an understanding would enhance the basic knowledge of dog biology, but also because a functional understanding of relationships between motor and visuospatial functions would enhance human abilities to improve canine training during different activities (animal-assisted therapy, police, guide for vision impaired). For example, it would be profitable to know the visuospatial orienting bias of a dog in order to optimize the capture of his attention during training or to choose the handling side that interferes less with the dog’s orienting attention.

## Materials and Methods

### Subjects

Subjects were 25 domestic dogs of various breeds (2 Australian Shepherds, 1 English Cocker Spaniel, 1 Flat-Coated Retriever, 1 Golden Retriever, 1 Beagle, 1 Shiba Inu, 1 Weimaraner, 17 mixed-breed dogs). Dogs ranged from 1 to 13 years of age (5.3 ± 3.8; mean ± s.d.). All dogs (16 females, 10 of which neutered; 9 males, 2 of which neutered) were pets living in households.

### Testing apparatus and procedure

The experiment was carried out in a large isolated room (20 m^2^) at the Department of Veterinary Medicine of Bari University, Italy.

**Figure 4:**
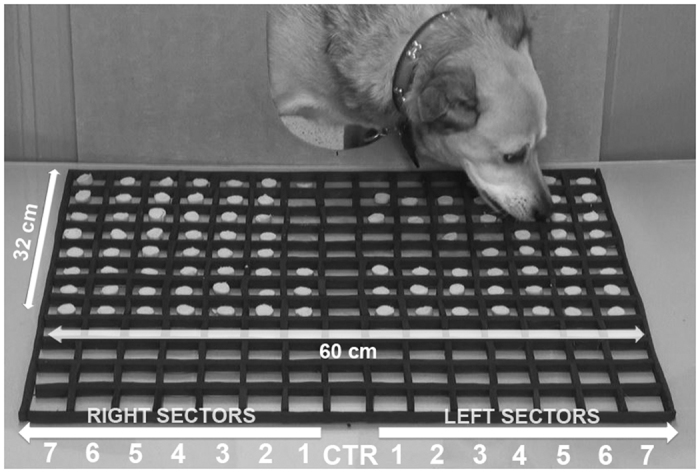
Testing apparatus used to study visuospatial bias in dogs. For the analysis, the Plexiglas surface was divided into an array of 15 identical vertical sectors (7 sectors to the right and to the left side of the central one “CTR”); explanation in the text.

The testing apparatus consisted of a Plexiglas board measuring 75 × 40 cm; the board was divided by rubber strips (1 cm in height) into 120 compartments, each one measuring 5 cm × 5 cm (15 sectors of 8 compartments each); each compartment was filled with a food item (a circular würstel slice), except the central sector (which was left empty), for a total of 112 food items (see Fig. 4). All slices were the same size and were placed in the middle of each compartment. The Plexiglas board was covered with brown paper on its lower surface and was fixed at a height of 34 cm from the floor. For small breed subjects, another Plexiglas board was built (60 cm × 32 cm; 16.5 cm above floor level with 4 cm × 4 cm compartments). The dogs could access the Plexiglas board by inserting their head through a U-shaped gap made in the centre of a wooden barrier, at about dog head height (see Fig. 4).

Trials were video-recorded with a high-resolution camera (Sony HDR-XR550) placed on a tripod at a distance of 1 m on the opposite side of the board from the dog and the whole experiment was monitored using a closed-circuit video system.

The test consisted of four trials (5 minutes each), during which the dogs were led on a long leash to the barrier and then left free to insert their head in the gap and to eat the food items put on the Plexiglas board. There was a 10 min interval between each trial. The owner was positioned on the dog side and behind the testing apparatus (standing motionless without saying or doing anything). Since the owner’s position in the dog’s right or left visual field can affect its emotional state, owner position with respect to dogs was alternated during the course of the trials^42^. We wished to avoided placing the owner on the same axis as the dog and the apparatus since there is clear evidence that dogs may be able to detect visuo-spatial information from their owners^43^,^44^ (e.g. by looking at human faces, dogs are able to recognize the direction in which humans are facing or gazing and their attentional states). At the same time, from a pilot study, we directly noted that when the owner was positioned central to and behind the dog, the latter was distracted (since the dog loses sight of its owner it frequently turns its head back in order to check the owner’s position). Each dog was tested twice (2 trials per session), and the second experimental session took place at least one week after the first (12.12 ± 3.08 days; mean ± S.D.). The left-right position of the owner with respect to dogs was counterbalanced across the whole sample. The owners were told not to interact in any way with the dogs.

### Paw preference test

Paw preference was determined using the most commonly used challenge to test canine motor preferences, i.e. the Kong ball test^26^. Each dog was tested 10 minutes after the end of each experimental session using a modified version of the Kong test used by Branson and Rogers, 2006. Depending on its weight, the subject was presented with a Large Classic Kong or with a Small Classic Kong, and was left free to play with it. The Kong was filled with the same würstels used during the trials and presented to the dogs on a flat surface in an empty room (15 m^2^) at the Department of Veterinary Medicine of Bari University. Dogs’ paw usage was recorded over a period of 15 minutes using a digital video camera by the same operator throughout the experiment, who was instructed not to interact with the dog during testing.

Pre-requisite for inclusion in the analysis was a minimum of 50 (left + right) paw attempts to stabilise the Kong in 60 min (15 min × 4 sessions).

### Data analysis

Individual lateralisation in paw usage during the Kong test was calculated using the following index (LI_paw-preference_): (Total number of times the left paw was used during the test)/(total number of times the left paw was used during the test + total number of times the right paw was used during the test)) × 100.

Animals were selected on the basis of a significant individual preference (estimated by one-tailed binomial test <0.05) for using a particular paw in the total number of attempts during the 15 min of testing.

In addition, visuospatial bias in orienting attention was computed using two laterality indices as follows:

LI _(Number of food items eaten)_ = (L − R/L + R) × 100, where L and R indicate, respectively, the mean number of food items eaten from the left and the right hemispace during the cancellation task.

LI _(Eating order of food items)_ = (L − R/L + R) × 100, where L and R indicate, respectively, the mean score obtained by the eating order of food items from the left and the right hemispace during the cancellation task.

In addition, since in dogs, as in other species, there is clear evidence of a right-to-left hemisphere dominance in taking charge of behavior when routine responses to stimuli emerge as a result of familiarization, the two laterality indices of visuospatial biases were also calculated for each session in order to verify whether the dogs were consistent in their performance across trials during the cancellation task.

Although none of the dogs were specifically food-deprived, most had not eaten for 8–10 hrs before testing. In addition, if within 2 minutes the dog did not start either to interact with the Kong ball or to eat from the Plexiglas board, the test was stopped and the subjects removed from the sample.

Parametric data were analyzed in a 2-within factors ANOVA model, considering as a first repeated measure variable the distance of each sector from the centre of each left/right position (7 in dogs), and, as a second factor, the difference between the amount of left and right food items eaten by each dog in each single position. Sex and paw preference were considered as the between-subjects factors.

The experiments were conducted according to the protocols approved by the Italian Ministry for Scientific Research in accordance with EC regulations and were approved by the Department of Veterinary Medicine (University of Bari) Ethics Committee EC (Approval Number: 7/15); in addition, before the experiment began, the procedure was explained to owners and written informed consent was obtained.

## Additional Information

**How to cite this article**: Siniscalchi, M. *et al*. Relationship between visuospatial attention and paw preference in dogs. *Sci. Rep*. **6**, 31682; doi: 10.1038/srep31682 (2016).

## Supplementary Material

Supplementary Information
