# Supplementary material for: Relationship between visuospatial attention and paw preference in dogs
Source: Sci Rep. 2016 Aug 22;6:31682. doi: 10.1038/srep31682 (PMC4992877; doi:10.1038/srep31682)
Supplement: Supplementary Information [file srep31682-s1.pdf]

Supplementary Information

Title of manuscript:

Relationship between visuospatial attention and paw preference in dogs

Authors:

Marcello Siniscalchi, Serenella d’Ingeo, Serena Fornelli and Angelo Quaranta

Supplementary information includes:

Supplementary analysis for the Kong ball test

Supplementary analysis

Kong ball test

Video footages from a sub sample of six dogs (three left preferent and three right preferent) were analysed in order to score the total number of ipsilateral (i.e. the Kong ball was on the same side of the body with respect to the paw used to stabilise it), contralateral (i.e. the Kong ball was on the contralateral side of the body with respect to the paw used to stabilise it) and frontal attempts to the Kong (the Kong and the longitudinal axis of the dog's body were all in a straight line). Overall, contralateral attempts (11.4 %) to the Kong occurred less frequently with respect to frontal (33.7 %) and ipsilateral ones (54.9 %). The lower frequency of contralateral attempts to stabilise the Kong ball could be explained by the fact that, differently from contralateral forelimb usage in humans, contralateral paw usage in dogs may seriously interfere with normal posture and, therefore, stability (i.e. contralateral paw usage has a cost in terms of dog's ergonomic movements). Nevertheless, the statistical analysis revealed a significant preference for using the preferred paw during contralateral ($t(5) = 5.707$, $P < 0.01$; one-tailed one-sample t-test) and frontal attempts ($t(5) = 5.577$, $P < 0.01$; one-tailed one-sample t-test) to stabilise the Kong ball.
